# Is the pan-genome also a pan-selectome?

**DOI:** 10.12688/f1000research.1-16.v1

**Published:** 2012-09-21

**Authors:** Francisco Rodriguez-Valera, David W Ussery

**Affiliations:** 1 Departmento de Producción Vegetal y Microbiología, Universidad Miguel Hernandez, San Juan de Alicante, 03550, Spain; 2 Center for Biological Sequence Analysis, Department of Systems Biology, The Technical University of Denmark, Kgs. Lyngby, Denmark

## Abstract

The comparative genomics of prokaryotes has shown the presence of conserved regions containing highly similar genes (the 'core genome') and other regions that vary in gene content (the ‘flexible’ regions). A significant part of the latter is involved in surface structures that are phage recognition targets. Another sizeable part provides for differences in niche exploitation. Metagenomic data indicates that natural populations of prokaryotes are composed of assemblages of clonal lineages or "meta-clones" that share a core of genes but contain a high diversity by varying the flexible component. This meta-clonal diversity is maintained by a collection of phages that equalize the populations by preventing any individual clonal lineage from hoarding common resources. Thus, this polyclonal assemblage and the phages preying upon them constitute natural selection units.

## The pan-genomic world

Bacterial and archaeal genomes show a surprising diversity in gene content even in otherwise very similar strains ^1, 2^. Some parts of the genome are shared and keep a high sequence similarity. The 95% average nucleotide identity (ANI) appears as a kind of “magic number” that fits the definition of most classical species, replacing the pre-genomic 70% DNA-DNA hybridization “golden rule” ^3^. This is the ‘core’ of prokaryotic genomes (surprisingly similar figures hold for Bacteria and Achaea in spite of their highly divergent molecular biology) ^4, 5^.

However, the most remarkable finding of prokaryotic genomes is the presence of other genomic regions that are extremely variable and differ in gene content and synteny from one strain to another ^6^. One consequence is that the diversity of genes found in a single prokaryotic species is stunning; for example in *Escherichia coli* with about 5000 genes per genome, it is estimated that there could be about 45,000 different gene families in its pan-genome ^7^. The ‘open-ness’ of a bacterial pan-genome for a species ranges from roughly twice the size of an individual genome, to more than ten-fold ^6, 8^. Thus, the genetic diversity hidden in the prokaryotic domain is much higher than initially suspected. This raises several questions regarding the biology and evolution of prokaryotic cells. How is this enormous diversity generated and, even more importantly, maintained? How does it impact an organism’s survival strategies and adaptation potential? These questions are fundamental gaps in our knowledge of the largest and oldest group of organisms on the planet.

The availability of multiple genomes from the same bacterial species has advanced greatly our knowledge of prokaryotic pangenomes ^2, 8^. Furthermore, the availability of large metagenomic datasets permits the analysis of the presence or absence of parts of the genomes of microbes that are known to be abundant in a specific habitat ^9– 12^; this bypasses the limitations and biases of pure culture retrieval of strains. We can begin to see general trends now in the pools of genes in the core and ‘flexible’ components of prokaryotic pan-genomes.

## The flexible pool and the cell surface

One major problem of the flexible pool is its remarkable diversity, which makes it hard to classify its genes. Being less widely distributed, they are more difficult to annotate and often appear as hypothetical proteins. However, as more genomes are sequenced, patterns start to be discernible. Particularly informative are the clusters of ‘flexible genes’ collected in genomic islands (GIs), which contain groups of contiguous genes, making functional inference much more reliable. Much of the flexible pool is collected in GIs of 10Kb or more. In this paper we will focus on some of these islands that are present in most (or all) strain genomes but containing different genes ( *i.e.* they are in the same genomic context and code for the same type of function or structure but the genes are only distantly related, if at all). For the sake of clarity we will designate these genomic islands found in many strains but containing different genes ‘flexible Genomic Islands’ (fGIs).

One kind of fGI that appears to have universal distribution encodes for the synthesis of exposed structures of the prokaryotic cell. One of the most remarkable examples of this phenomenon is the gene cluster that codes for the synthesis of the O-chain of the Gram-negative lipopolysaccharide (LPS; ^13^). Classically known as the ‘O-antigen’, the diversity of this exposed envelope in *Salmonella* or *Escherichia* has been known for many years ^14, 15^. The O-chain is a repeat-unit polysaccharide, the monomeric repeat has generally between two and six sugar residues. O-chains are extremely variable in the nature, order and linkage of the different sugars ^13, 16^. This complex polysaccharide is very important for the survival of the cell, providing the appropriate hydrophilic envelope to allow nutrient imports towards the cell ^17^. After subculture in the laboratory many strains lose part of the polysaccharide, originating “rough” mutants, which probably have diluted the critical importance that it had for the cell’s lifestyle. However, the importance of the O-chain for antibiotic susceptibility is long known, illustrating how the permeability properties of the cell vary with small changes affecting this structure ^18^. Further, the gene cluster for such an important cell component is extremely variable. This diversity has classically been explained by the advantage of the concomitant antigenic variation that could prevent the host from identifying, and eventually expelling, all the strains of one of these species. However, even accepting this simplistic inference from host-microbiome interaction, the variability found in free-living bacteria is comparable (if not higher) than those of specialized pathogens or symbionts ^17^.

**Figure 1.**
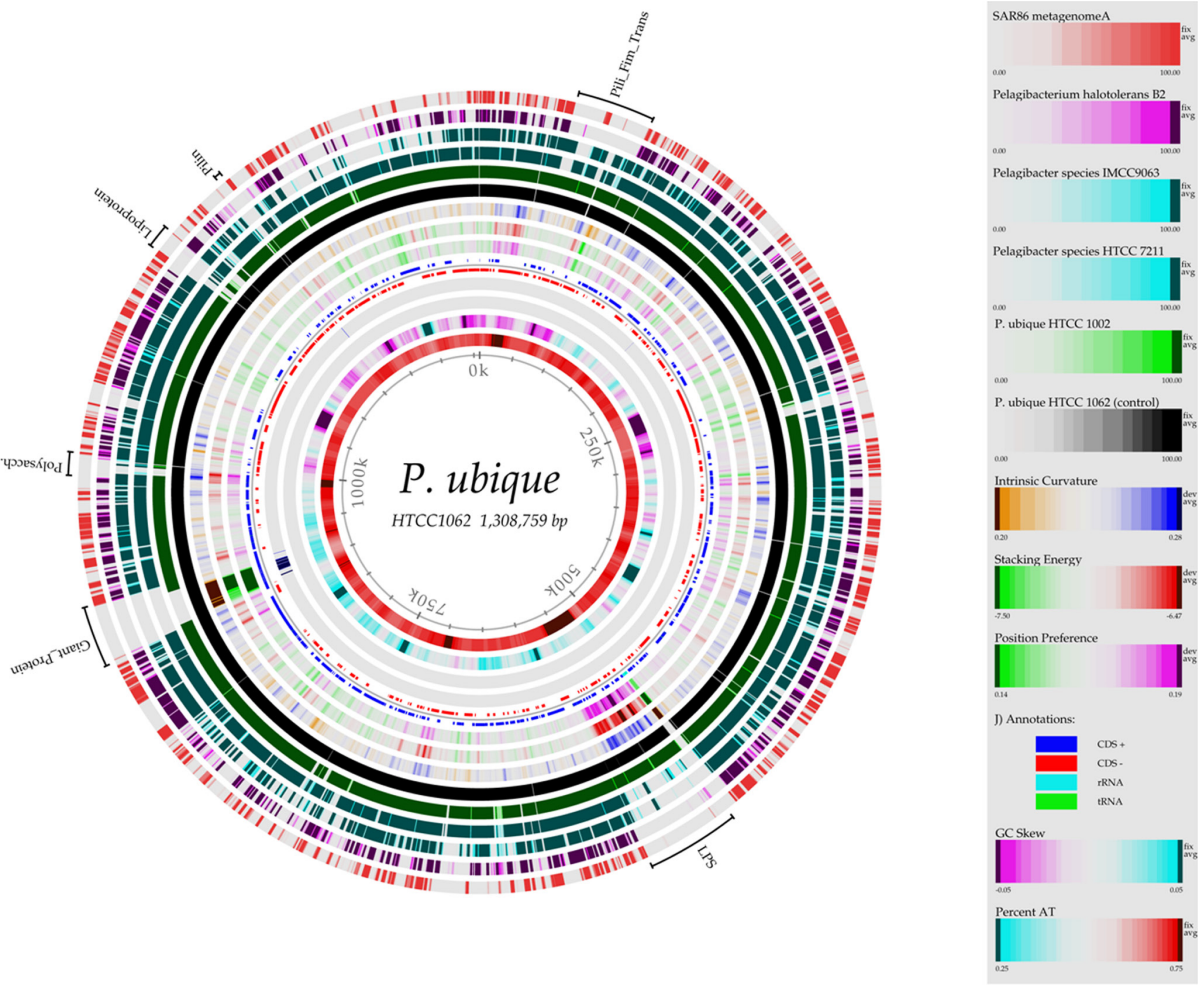
BLASTatlas for *Pelagibacter ubique*, showing fGIs along the chromosome.

As an example, Figure 1 shows the fGIs detected in the genomes available of Candidatus *Pelagibacter ubique*, probably the most abundant pelagic marine microbe. Further, in addition to the O-chain gene cluster, all known exposed structural motives that can vary reflect similar genomic patterns of variation. For example, capsular or slime layer polysaccharides ^5, 19^, the teichoic acids of Gram-positives ^20^, or the S-layer glycoproteins of archaea ^4, 21^ all seem to be located in fGIs. Other exposed structures that are also typical components of the flexible gene pool are flagella, pili and porins. An alternative way to view the diversity found in all these cell components is that they all are important phage recognition targets.

## Phages, phages everywhere

Viruses and their hosts are extremely entangled entities. In many environments there are on average about 10 phages for every one bacterium ^22^, which means that bacteria are under constant attack. There are millions of viruses in every drop of ocean water; on average, it is estimated that about a mole of viruses (6x10 ^23^) attack bacteria every minute in the oceans ^5, 6^. Some estimates suggest that a quarter of newly photosynthesized carbon in marine environments travels through the ‘‘viral shunt’’, moving it directly to dissolved organic carbon before grazers or other consumers can access it ^6, 7^. The diversity of bacteriophages is quite large, and dynamic, changing with time in a given environment ^23^. Presumably, this change in virus diversity reflects changes in bacterial diversity, since phages are obligate parasites, and many phages can only infect specific bacterial strains. It has been estimated that 60–70% of the bacterial genomes sequenced to date contain prophages ^8, 9^ (Rob Edwards, Personal communication). About two-thirds of all sequenced proteobacteria (no 'l') Gamma-proteobacterial and low GC Gram-positive bacteria harbor prophages ^10^; thus the phages are also part of the pan-genome for many of these organisms.

The surface of a cell is what is presented to the world (both friend and foe alike); in environments where bacteria are under constant attack from viruses, the need to often change the shape and appearance of surface proteins is compelling. Thus a good evolutionary strategy would be to vary these proteins, both by changing their structures, but also by distributing them amongst other bacteria within the population, where possible.

Can the need to diversify phage receptors explain the enormous diversity of the pan-genome? Certainly not, but it could be responsible for a large part. However, the other major component of the flexible pool might also be preserved via phage predation ^12^.

## The flexible pool and niche partitioning

Many components of the flexible pool are involved in niche partitioning: 1) transport of substrates and the cognate metabolic pathways required for their assimilation by the cell, 2) regulation, such as two component systems involved in fine tuning the response to environmental stimuli 3) respiratory chains and or protective mechanisms involved in different oxygen or light relationships. This has been found to apply to both bacteria ^11, 24– 26, 27^ and archaea ^4^. For heterotrophic osmotrophs the transporter baggage carried in the genome is determined largely by lifestyle and niche specialization. Accordingly the transporters found in different clonal lineages are extremely variable and are typical components of the flexible gene pool. In a remarkably parallel way, *hli* genes (coding for high-light inducible proteins) present in marine picocyanobacteria might influence the fine light qualities exploited by these widespread phototrophs and are also typical components of the flexible pool of these microbes ^28^. A similar story is depicted by the tonB receptors involved in the transport of micronutrients ^29^.

It is important to emphasize here that fGIs related to phage sensitivity such as the O-chain of the LPS and fGIs related to niche specialization are genetically linked in a single replicon so that negative selection by phage predation would compensate automatically positive selection by overly efficient exploitation of resources. For example, a sudden increase in the concentration of nutrients that are exploited by a clone that might lead to a major clonal expansion would be kept in check by the increase in the concentration of the linked phage receptors ^12^. This mechanism of population control although negative for the short term prevalence of the clone might be good for its long term survival since it maintains the complexity of the community and its endurance.

## The pan-selectome

The unit of selection has been a major conundrum in evolutionary biology ^30^. Historically the proposals have been, according to times and fashions, going from the gene ^31^ to the community ^32^ or even the planet ^33^. In 1997 Ernst Mayr defined the unit of selection as “a discrete entity and a cohesive whole, an individual or a social group, the survival and successful reproduction of which is favored by selection owing to its possession of certain properties” ^34^.

**Figure 2.**
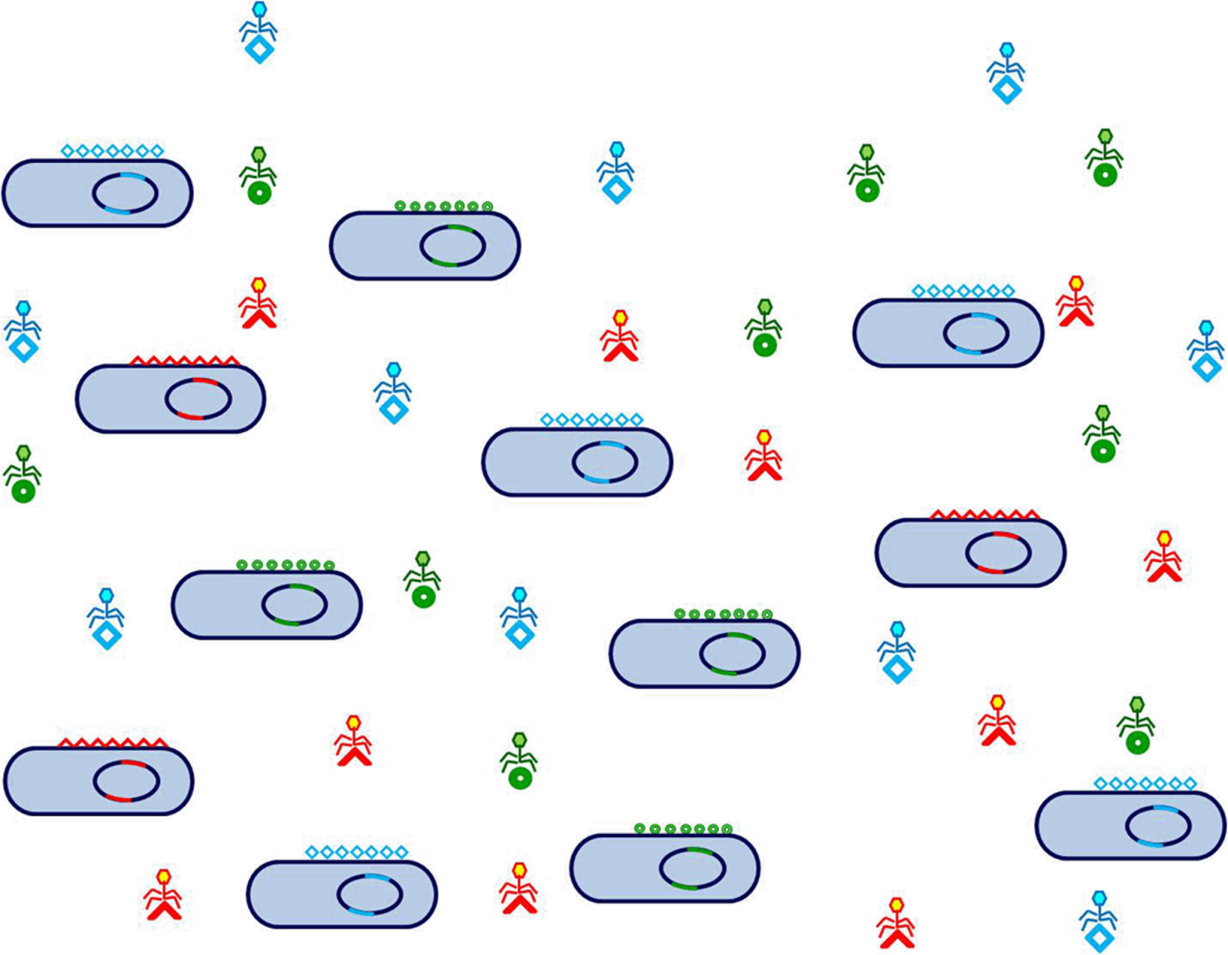
A prokaryotic population with cells and phages, as depicted by the Constant-Diversity model. The genome is indicated as a solid blue circle (core) with two flexible genome islands fGIs (see text) in different colors to indicate different genes but coding for the same functions. The two fGIs indicated code for the O-chain of the lipopolysaccharide (in a Gram negative bacterium) and for a set of transporters. In reality there are many more fGIs (often four or five or more); further many differential transporters or other niche exploitation related features can be coded in small flexible islands or islets interspersed along the core, but they are all genetically linked to the O-chain and other surface related fGIs that are major recognition targets for phages. Three types of phages and receptors have been indicated, by different geometric forms. This set of cellular clones and phages is in equilibrium since the disproportionate increase of cells or phages is prevented by the density dependent kinetics of phage infection. For example if one clone increases over a certain threshold it will be over-preyed by its phages, until population returns to normal following a classical Lotka-Volterra predator/prey equilibrium. This is favorable for the meta-clonal population, since it keeps different lineages with complementary ecological skills acting in tune. This meta-clonal/viral population can be selected as an unit to exploit similar environments such as the water column in the ocean that is very similar worldwide.

We would like to advance here the idea that in nature, the selection unit in a prokaryotic community or assemblage is an ensemble of clonal lineages that share the same (or highly similar) core genome but differ in the flexible gene complements regions ( *i.e.* the selection unit at the genomic level would be the pan-genome) Box 1. These meta-clonal populations are maintained and equalized by phage predation ^12^ in an analogous way as the immune system in a mammal maintains in check tumors. Thus, phage populations should be considered as belonging to the same selection unit, not only because they are part of the pan-genome (which they often are) but because they are instrumental for its long term preservation ( Figure 2). Furthermore, the different clonal lineages as retrieved by pure culture have little chance of succeeding in nature (or in complex biotechnological processes such as dairy or wine production or sewage treatment).

Although this idea invokes elements of group selection, a controversial theory of evolutionary biology ^35^, it is being proposed to explain evolution of prokaryotic populations, about which very little evolutionary theory has been solidly established, particularly outside of the walls of the test tube.

## Data supporting group selection in prokaryotes

One of us proposed a Constant-Diversity model based on the distribution of gene functions in the genomic islands that under-recruit in metagenomes, where the core genome recruits at high similarity ^12^. Since then, several independent workers have found data supporting this notion.

One of the most convincing demonstrations that different fGIs in prokaryotes are largely involved in phage sensitivity is the work of Avrani *et al.* ^36^, in which the resistance to phages in several isolates of *Prochlorococcus* could all be assigned to mutations taking place in Genomic Island 4 of this microbe identified previously ^9^, as involved in the synthesis of the O-chain of the lipopolysaccharide. By measuring the frequencies of mutants in metagenomic datasets the authors conclude: “abundant *Prochlorococcus* populations belonging to a single ecotype with common physiological and ecological characteristics are actually an assortment of subpopulations with different susceptibilities to co-occurring phages” and “Thus, large numbers of taxonomically identical organisms, fulfilling the same ecological role, are probably maintained in the environment as a result of micro-diversity in phage susceptibility regions” ^36^. A similar situation is found in *Synechococcus* where also resistant mutants were found to have altered genes in the O-chain region ^37^.

Many other recent developments support the coexistence of complex populations of phages and their hosts in natural communities ^23, 38– 41^. There is also evidence that phages contribute to keep high levels of host diversity ^42^ and that diversity promotes productivity ^43^.

Recently there have been other alternatives used to explain the diversity of pan-genomes by some type of kin selection, such as the so-called Black Queen hypothesis ^44^ in which some genes present in certain lineages can supply the functions for other clonal lineages. Along the same lines, Teusink *et al.* describe a “game theory” explanation for the coexistence of proteolytic and non-proteolytic strains in dairy multi-starter cultures ^45^. However, these models only make even more critical the role of phages to keep the proper ratios among the different cooperating lineages.

## Conclusion

We are proposing here a way of thinking about prokaryotic communities in which cells with a core above 95% ANI, but with a wide diversity of flexible genome complement, and phages praying on them, form an evolutionary selection unit, the “prokaryotic selecton”. This has important repercussions for the evolutionary biology of prokaryotes.

Box 1. **The pan-selectome and the evolutionary unit (a.k.a. species).**

The species as an evolutionary unit is at the centre of the modern Neo-Darwinian synthesis. Species are not just considered as a taxonomic level of classification but as a kind of biological entity or level of organization beyond the cell or the individual ^46^. However, this “natural species concept” has been difficult to transfer to prokaryotes due to the lack of sexual reproduction and the unpredictable levels of recombination (particularly when including the illegitimate one) of prokaryotic genomes ^47^. If the pan-selectomes described here are the units of selection, they might also fulfill the requirements to be considered natural evolutionary units. However, this requires a mechanism that provides the discontinuities in genetic diversity found in nature. Metagenomic data show that there are discontinuities located at *ca* 95% ANI, beyond which a gap indicates an empty space in the sequence diversity space ^48^. Of course this only applies to the core genome of the meta-clones but still reflects a coherence that requires an evolutionary drive reminiscent of the breeding barriers found in animal species for example. How could the meta-clones explain such discontinuities? This critical question remains to be answered. However, we would like to advance one hypothesis that we call “the maverick hypothesis”. Let’s assume that a meta-clone of bacteria and phages is established somewhere, for example, exploiting the degradation of chitin, a common component in the water column of the ocean. A pan-genome evolves that allows for the efficient exploitation of the multiple resources associated to this polysaccharide (other accompanying organic molecules, phosphorus and nitrogen source etc.). The populations of this microbe have also a complement of phages to keep a well-equilibrated consortium. The physical proximity, near where the resources (such as zooplankton remains) are found facilitates genomic homogenization by homologous recombination. The pools of genes in the flexible genome can diverge enormously but the core will remain relatively coherent. The rise of a “maverick” that would try to form a monoclonal population diverging away from the homogenizing influence of the rest would be prevented by the excessive phage predation pressure coupled to less efficient exploitation of resources. This trend in the long run might be enough to provide the discontinuities required to form a natural species-like entity.

## References

[1] Passerini D Beltramo C Coddeville M: Genes but not genomes reveal bacterial domestication of Lactococcus lactis. *PLoS One.* 2010;5(12):e15306 10.1371/journal.pone.0015306 21179431 PMC3003715

[2] Muzzi A Donati C: Population genetics and evolution of the pan-genome of Streptococcus pneumoniae. *Int J Med Microbiol.* 2011;301(8):619–622 10.1016/j.ijmm.2011.09.008 22000739

[3] Konstantinidis KT Tiedje JM: Towards a genome-based taxonomy for prokaryotes. *J Bacteriol.* 2005;187(18):6258–6264 10.1128/JB.187.18.6258-6264.2005 16159757 PMC1236649

[4] Cuadros-Orellana S Martin-Cuadrado AB Legault B: Genomic plasticity in prokaryotes: the case of the square haloarchaeon. *ISME J.* 2007;1(3):235–245 10.1038/ismej.2007.35 18043634

[5] Pasic L Rodriguez-Mueller B Martin-Cuadrado AB: Metagenomic islands of hyperhalophiles: the case of Salinibacter ruber. *BMC Genomics.* 2009;10:570 10.1186/1471-2164-10-570 19951421 PMC2800850

[6] Tettelin H Masignani V Cieslewicz MJ: Genome analysis of multiple pathogenic isolates of Streptococcus agalactiae: implications for the microbial "pan-genome". *Proc Natl Acad Sci U S A.* 2005;102(39):13950–13955 10.1073/pnas.0506758102 16172379 PMC1216834

[7] Lukjancenko O Wassenaar TM Ussery DW: Comparison of 61 sequenced Escherichia coli genomes. *Microb Ecol.* 2010;60(4):708–720 10.1007/s00248-010-9717-3 20623278 PMC2974192

[8] Snipen L Almoy T Ussery DW: Microbial comparative pan-genomics using binomial mixture models. *BMC Genomics.* 2009;10:385 10.1186/1471-2164-10-385 19691844 PMC2907702

[9] Coleman ML Sullivan MB Martiny AC: Genomic islands and the ecology and evolution of Prochlorococcus. *Science.* 2006;311(5768):1768–1770 10.1126/science.1122050 16556843

[10] Legault BA Lopez-Lopez A Alba-Casado JC: Environmental genomics of "Haloquadratum walsbyi" in a saltern crystallizer indicates a large pool of accessory genes in an otherwise coherent species. *BMC Genomics.* 2006;7:171 10.1186/1471-2164-7-171 16820057 PMC1560387

[11] Wilhelm LJ Tripp HJ Givan SA: Natural variation in SAR11 marine bacterioplankton genomes inferred from metagenomic data. *Biol Direct.* 2007;2:27 10.1186/1745-6150-2-27 17988398 PMC2217521

[12] Rodriguez-Valera F Martin-Cuadrado AB Rodriguez-Brito B: Explaining microbial population genomics through phage predation. *Nat Rev Microbiol.* 2009;7(11):828–836 10.1038/nrmicro2235 19834481

[13] Samuel G Reeves P: Biosynthesis of O-antigens: genes and pathways involved in nucleotide sugar precursor synthesis and O-antigen assembly. *Carbohydr Res.* 2003;338(23):2503–2519 10.1016/j.carres.2003.07.009 14670712

[14] Roantree RJ: Salmonella O antigens and virulence. *Annu Rev Microbiol.* 1967;21:443–466 10.1146/annurev.mi.21.100167.002303 4860265

[15] Reeves P: Evolution of Salmonella O antigen variation by interspecific gene transfer on a large scale. *Trends Genet.* 1993;9(1):17–22 10.1016/0168-9525(93)90067-R 8434412

[16] Aydanian A Tang L Morris JG: Genetic diversity of O-antigen biosynthesis regions in Vibrio cholerae. *Appl Environ Microbiol.* 2011;77(7):2247–2253 10.1128/AEM.01663-10 21317260 PMC3067440

[17] Nazarenko EL Crawford RJ Ivanova EP: The structural diversity of carbohydrate antigens of selected gram-negative marine bacteria. *Mar Drugs.* 2011;9(10):1914–1954 10.3390/md9101914 22073003 PMC3210612

[18] Yethon JA Gunn JS Ernst RK: Salmonella enterica serovar typhimurium waaP mutants show increased susceptibility to polymyxin and loss of virulence *In vivo*. *Infect Immun.* 2000;68(8):4485–4491 10.1128/IAI.68.8.4485-4491.2000 10899846 PMC98355

[19] Schaffer C Messner P: Glycobiology of surface layer proteins. *Biochimie.* 2001;83(7):591–599 10.1016/S0300-9084(01)01299-8 11522387

[20] Belda-Ferre P Cabrera-Rubio R Moya A: Mining virulence genes using metagenomics. *PLoS One.* 2011;6(10):e24975 10.1371/journal.pone.0024975 22039404 PMC3198465

[21] Hansen EE Lozupone CA Rey FE: Pan-genome of the dominant human gut-associated archaeon, Methanobrevibacter smithii, studied in twins. *Proc Natl Acad Sci U S A.* 2011;108(Suppl 1):4599–4606 10.1073/pnas.1000071108 21317366 PMC3063581

[22] Breitbart M: Marine viruses: truth or dare. *Ann Rev Mar Sci.* 2012;4:425–448 10.1146/annurev-marine-120709-142805 22457982

[23] Rodriguez-Brito B Li L Wegley L: Viral and microbial community dynamics in four aquatic environments. *ISME J.* 2010;4(6):739–751 10.1038/ismej.2010.1 20147985

[24] Ivars-Martinez E Martin-Cuadrado AB D'Auria G: Comparative genomics of two ecotypes of the marine planktonic copiotroph Alteromonas macleodii suggests alternative lifestyles associated with different kinds of particulate organic matter. *ISME J.* 2008;2(12):1194–1212 10.1038/ismej.2008.74 18670397

[25] Bon E Delaherche A Bilhere E: Oenococcus oeni genome plasticity is associated with fitness. *Appl Environ Microbiol.* 2009;75(7):2079–2090 10.1128/AEM.02194-08 19218413 PMC2663225

[26] Siezen RJ Tzeneva VA Castioni A: Phenotypic and genomic diversity of Lactobacillus plantarum strains isolated from various environmental niches. *Environ Microbiol.* 2010;12(3):758–773 10.1111/j.1462-2920.2009.02119.x 20002138

[27] Snitkin ES Zelazny AM Montero CI: Genome-wide recombination drives diversification of epidemic strains of Acinetobacter baumannii. *Proc Natl Acad Sci U S A.* 2011;108(33):13758–13763 10.1073/pnas.1104404108 21825119 PMC3158218

[28] Kettler GC Martiny AC Huang K: Patterns and implications of gene gain and loss in the evolution of Prochlorococcus. *PLoS Genet.* 2007;3(12):e231 10.1371/journal.pgen.0030231 18159947 PMC2151091

[29] Mirus O Strauss S Nicolaisen K: TonB-dependent transporters and their occurrence in cyanobacteria. *BMC Biol.* 2009;7:68 10.1186/1741-7007-7-68 19821963 PMC2771747

[30] Aakra A Nyquist OL Snipen L: Survey of genomic diversity among Enterococcus faecalis strains by microarray-based comparative genomic hybridization. *Appl Environ Microbiol.* 2007;73(7):2207–2217 10.1128/AEM.01599-06 17220255 PMC1855650

[31] Clegg MT Allard RW Kahler AL: Is the gene the unit of selection? Evidence from two experimental plant populations. *Proc Natl Acad Sci U S A.* 1972;69(9):2474–2478 10.1073/pnas.69.9.2474 4506768 PMC426968

[32] Day MD Beck D Foster JA: Microbial Communities as Experimental Units. *Bioscience.* 2011;61(5):398–406 10.1525/bio.2011.61.5.9 21731083 PMC3128510

[33] Sugimoto T: Darwinian evolution does not rule out the gaia hypothesis. *J Theor Biol.* 2002;218(4):447–455 10.1006/jtbi.2002.3091 12384048

[34] Mayr E: The objects of selection. *Proc Natl Acad Sci U S A.* 1997;94(6):2091–2094 10.1073/pnas.94.6.2091 9122151 PMC33654

[35] Borrello ME: The rise, fall and resurrection of group selection. *Endeavour.* 2005;29(1):43–47 10.1016/j.endeavour.2004.11.003 15749153

[36] Avrani S Wurtzel O Sharon I: Genomic island variability facilitates Prochlorococcus-virus coexistence. *Nature.* 2011;474(7353):604–608 10.1038/nature10172 21720364

[37] Marston MF Pierciey FJ Jr Shepard A: Rapid diversification of coevolving marine Synechococcus and a virus. *Proc Natl Acad Sci U S A.* 2012;109(12):4544–4549 10.1073/pnas.1120310109 22388749 PMC3311363

[38] Menge DN Weitz JS: Dangerous nutrients: evolution of phytoplankton resource uptake subject to virus attack. *J Theor Biol.* 2009;257(1):104–115 10.1016/j.jtbi.2008.10.032 19068219

[39] Kashiwagi A Yomo T: Ongoing phenotypic and genomic changes in experimental coevolution of RNA bacteriophage Qbeta and Escherichia coli. *PLoS Genet.* 2011;7(8):e1002188 10.1371/journal.pgen.1002188 21829387 PMC3150450

[40] Shapiro OH Kushmaro A Brenner A: Bacteriophage predation regulates microbial abundance and diversity in a full-scale bioreactor treating industrial wastewater. *ISME J.* 2010;4(3):327–336 10.1038/ismej.2009.118 19924159

[41] Albertsen M Hansen LB Saunders AM: A metagenome of a full-scale microbial community carrying out enhanced biological phosphorus removal. *ISME J.* 2012;6(6):1094–1106 10.1038/ismej.2011.176 22170425 PMC3358022

[42] Paterson S Vogwill T Buckling A: Antagonistic coevolution accelerates molecular evolution. *Nature.* 2010;464(7286):275–278 10.1038/nature08798 20182425 PMC3717453

[43] Cardinale BJ: Biodiversity improves water quality through niche partitioning. *Nature.* 2011;472(7341):86–89 10.1038/nature09904 21475199

[44] Morris JJ Lenski RE Zinser ER: The Black Queen Hypothesis: evolution of dependencies through adaptive gene loss. *MBio.* 2012;3(2):e00036–12 10.1128/mBio.00036-12 22448042 PMC3315703

[45] Teusink B Bachmann H Molenaar D: Systems biology of lactic acid bacteria: a critical review. *Microb Cell Fact.* 2011;10(Suppl 1):S11 10.1186/1475-2859-10-S1-S11 21995498 PMC3231918

[46] de Queiroz K: Ernst Mayr and the modern concept of species. *Proc Natl Acad Sci U S A.* 2005;102(Suppl 1):6600–6607 10.1073/pnas.0502030102 15851674 PMC1131873

[47] Doolittle WF: Population genomics: how bacterial species form and why they don't exist. *Curr Biol.* 2012;22(11):R451–453 10.1016/j.cub.2012.04.034 22677288

[48] D Caro-Quintero A Konstantinidis KT: Bacterial species may exist, metagenomics reveal. *Environ Microbiol.* 2012;14(2):347–355 10.1111/j.1462-2920.2011.02668.x 22151572

