# Supplementary material for: Is the pan-genome also a pan-selectome?
Source: F1000Res. 2012 Sep 21;1:16. [Version 1] doi: 10.12688/f1000research.1-16.v1 (PMC3782348; doi:10.12688/f1000research.1-16.v1)
Supplement: Peer review history

# Peer review: Referee response for version 1

**Published:** 2012-10-05

**Authors:** Granger Sutton

**Affiliations:** 1 J. Craig Venter Institute, 9704 Medical Center Drive, Rockville, MD 20850, USA

I work on pan-genome analysis tools and found the article well written and interesting.

I have read this submission. I believe that I have an appropriate level of expertise to confirm that it is of an acceptable scientific standard.

# Peer review: Referee response for version 1

**Published:** 2012-09-28

**Authors:** Ben Busby, Michael Galperin

**Affiliations:** 1 The School of Biochemistry, NCBI/NLM/NIH, Bethesda, MD, USA; 2 National Center for Biotechnology Information (NCBI), National Institutes of Health (NIH), Bethesda, MD, USA

This is a clearly written and interesting opinion that we mostly agree with.

Together with the recent studies of the CRISPR system and Patrick Forterre’s just-published provocative concept of the “virocell”, this paper reflects – and promotes – a growing appreciation of the importance of bacteriophages in shaping the bacterial (prokaryotic) world. While we believe that the relationships between bacteria and their phages are even more complex than discussed here (e.g. phage lysogeny likely plays a greater and more complex role) this manuscript is definitely a good step in the right direction.

We have read this submission. We believe that we have an appropriate level of expertise to confirm that it is of an acceptable scientific standard.
